# Supplementary figures and images for: Heparin-enriched plasma proteome is significantly altered in Alzheimer’s disease
Source: Mol Neurodegener. 2024 Oct 8;19:67. doi: 10.1186/s13024-024-00757-1 (PMC11460197; doi:10.1186/s13024-024-00757-1)

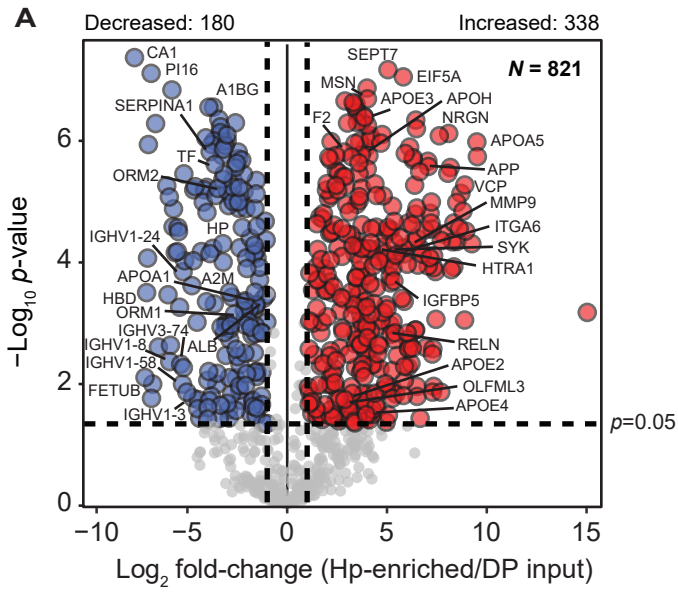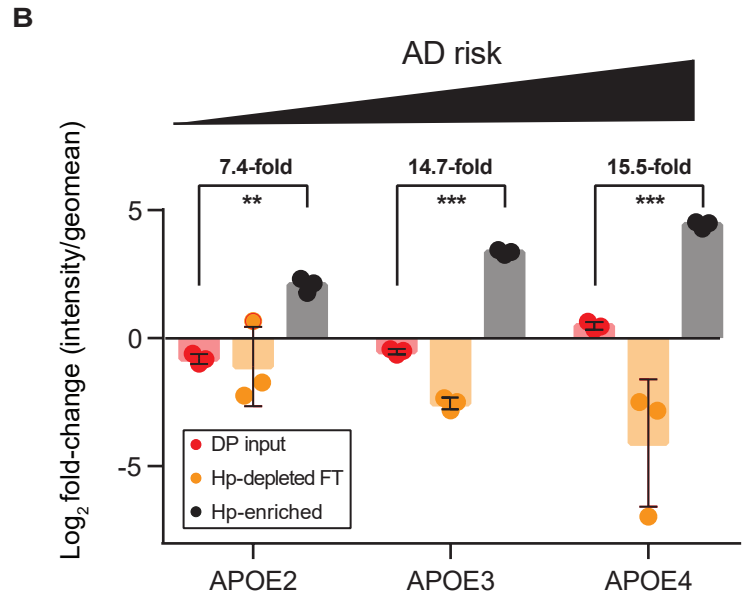

Supplement: Supplementary file 1 — Additional file 1: Supplemental Figure 1. Comparing heparin enrichment across APOE isoforms.A)Thevolcano plot shows differentially enriched proteins in the Hp-enriched fractions (n = 3) of pooled plasma sample compared to the DP inputs (n = 3). Red circles represent significant Hp-enriched proteins in plasma and examples of AD-related HBPs are highlighted, in addition to APOE isoforms. Blue symbols represent proteins significantly depleted from the Hp-enriched fractions. The significance cutoff is p < 0.05 (ANOVA with Tukey post-hoc correction) and fold-change > 2.B)The bar plot shows average abundance changes of APOE2, APOE3 and APOE4 isoforms across three replicates within each fraction of pooled plasma (DP input = 3, Hp-depleted FT = 3, Hp-enriched = 3). The y-axis is calculated by average log2 fold-change of protein intensity from three replicates over their geomean across all 9 samples. The significance of difference was determined by ANOVA with Tukey post-hoc correction and denoted with stars (* p < 0.05, ** p < 0.01, *** p < 0.001). [file 13024_2024_757_MOESM1_ESM.pdf]

**A**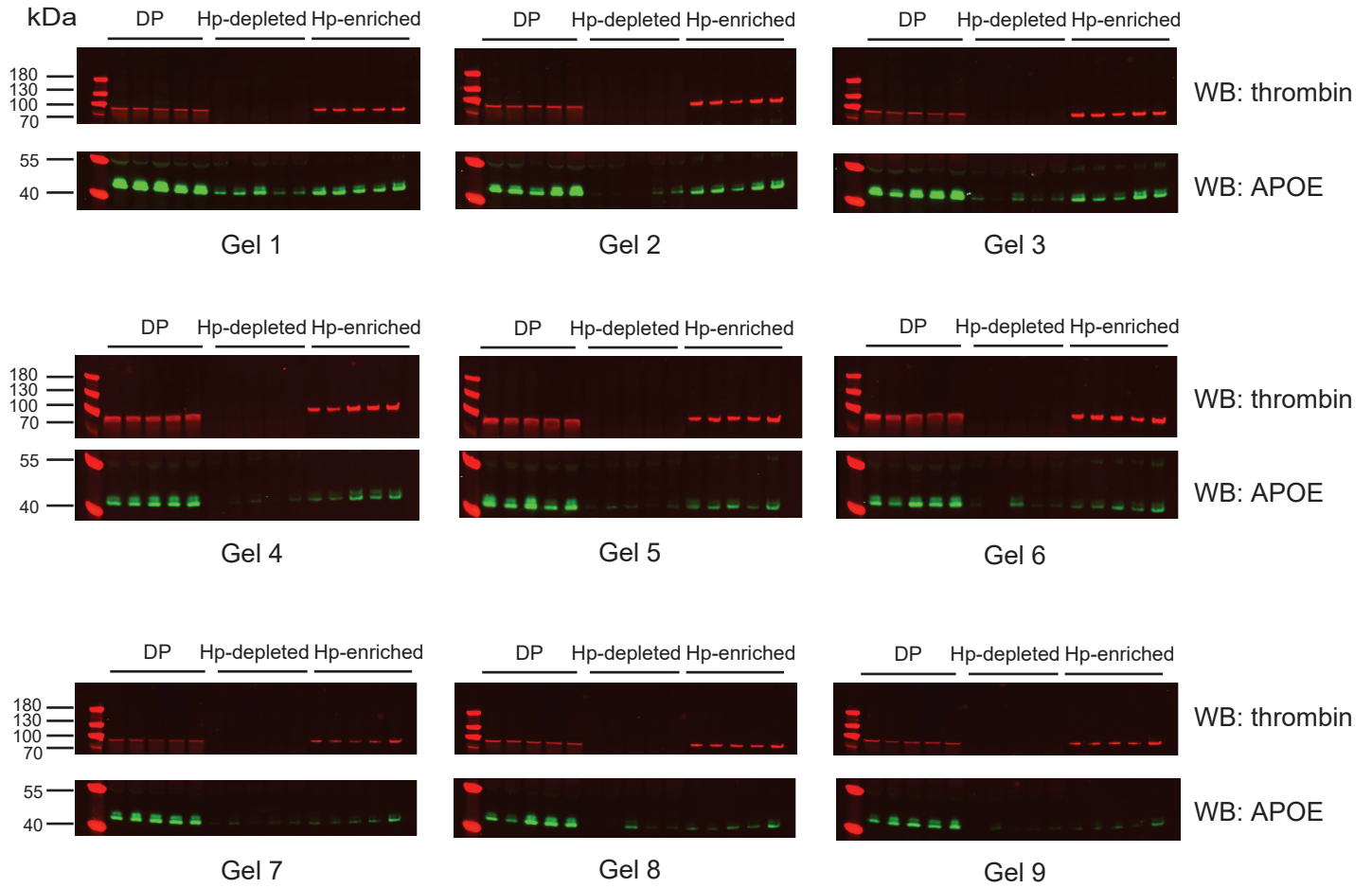

Supplement: Supplementary file 2 — Additional file 2: Supplemental Figure 2. Western blotting of thrombin and APOE in Set 1 (n = 36). A) Western blotting was conducted on DP input, Hp-depleted FT, and Hp-enriched fraction obtained from Set 1 samples, which included 18 control and 18 AD cases. Thrombin (~75 kDa) and APOE (~34 kDa) were the target HBPs of interest. On each gel, 2 control samples, 2 AD samples, and 1 GPS sample (see method) were loaded for each fraction. The results show that both thrombin and APOE were depleted from the DP input and Hp-depleted FT and enriched in the Hp-enriched fraction. WB: western blotting. [file 13024_2024_757_MOESM2_ESM.pdf]

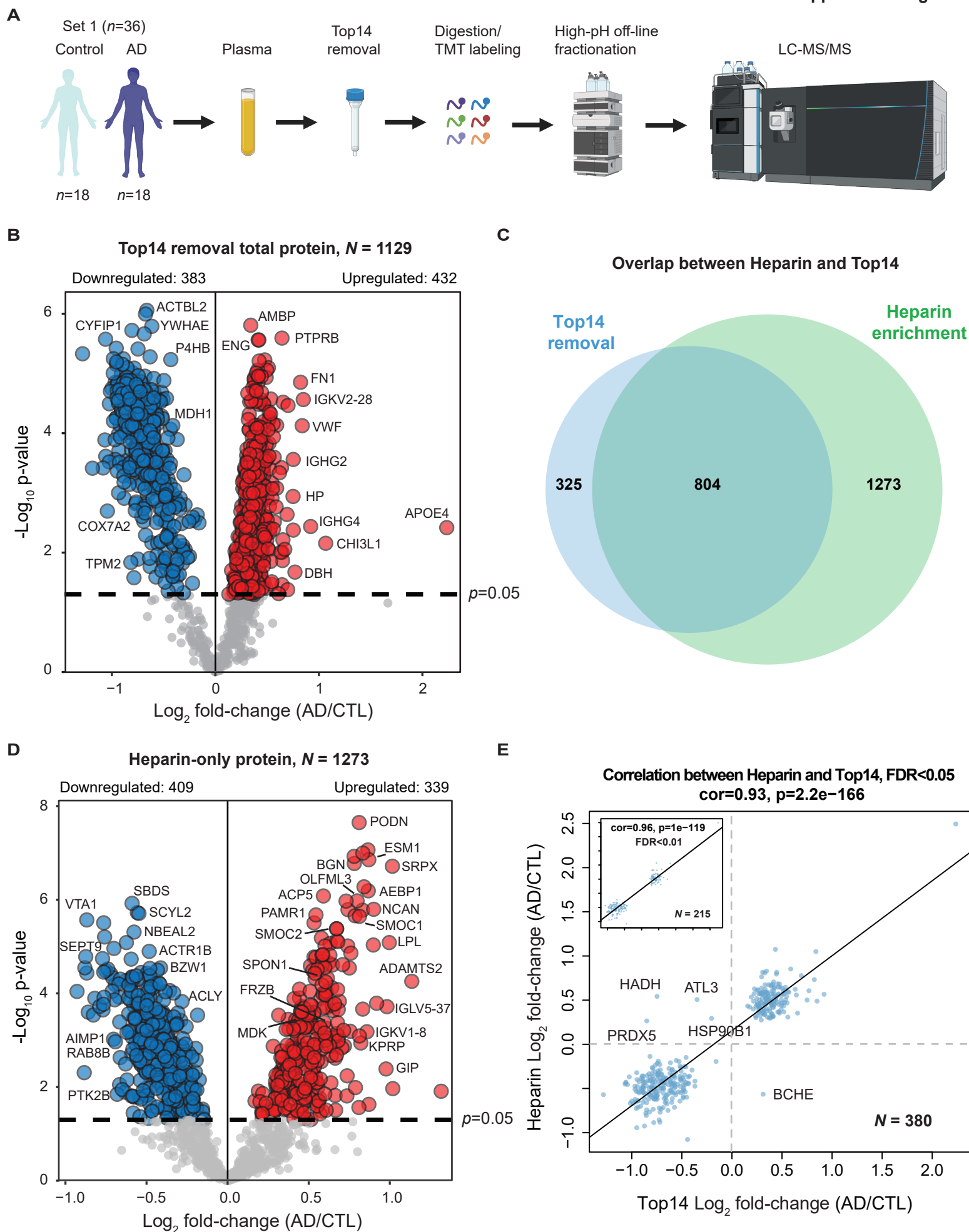

Supplement: Supplementary file 3 — Additional file 3: Supplemental Figure 3. Comparison of immunodepleted and heparin-enriched plasma proteomes. A) The Set 1 (control = 18, AD = 18) underwent top 14 removal and the same TMT-MS analysis as performed on the Hp-enriched Set 1. B) The volcano plot illustrates the differential abundance of 1,129 proteins between the control and AD groups. The x-axis represents the log2 fold-change (AD vs CTL), while the y-axis represents the Student’s t-statistic (-log10 p-value) calculated for all proteins in the pairwise comparison. Proteins significantly increased in AD (N = 432) are highlighted in red (p < 0.05), whereas those significantly decreased in AD (N = 383) are depicted in blue. Grey dots represent proteins with insignificant changes. C) The number and overlap of proteins quantified in the top 14 (N = 1,129) and Hp-enriched (N = 2,077) datasets with less than 50% missing values are shown. There are 1,273 proteins only detected by heparin enrichment, while the top 14 method identifies only 325 specific proteins. D) The volcano plot illustrates the differential abundance of 1,273 proteins (AD vs Control) uniquely identified in the Hp-enriched dataset. The x-axis represents the log2 fold-change (AD vs CTL), while the y-axis represents the Student’s t-statistic (-log10 p-value) calculated for all proteins in the pairwise comparison. Proteins significantly increased in AD (N = 339) are highlighted in red (p < 0.05), whereas those significantly decreased in AD (N = 409) are depicted in blue. Grey dots represent proteins with insignificant changes. E) A scatter plot shows the Pearson correlation between log2 fold-change (AD vs CTL) of significantly altered proteins in both the top 14 and Hp-enriched datasets. There are 380 proteins overlapping between the two sets, showing significant changes in AD with a BH FDR-corrected p-value< 0.05 in both datasets. Only 5 out of 380 proteins exhibited discordant changes in AD vs Control, demonstrating a high degree of concordan [file 13024_2024_757_MOESM3_ESM.pdf]

**A**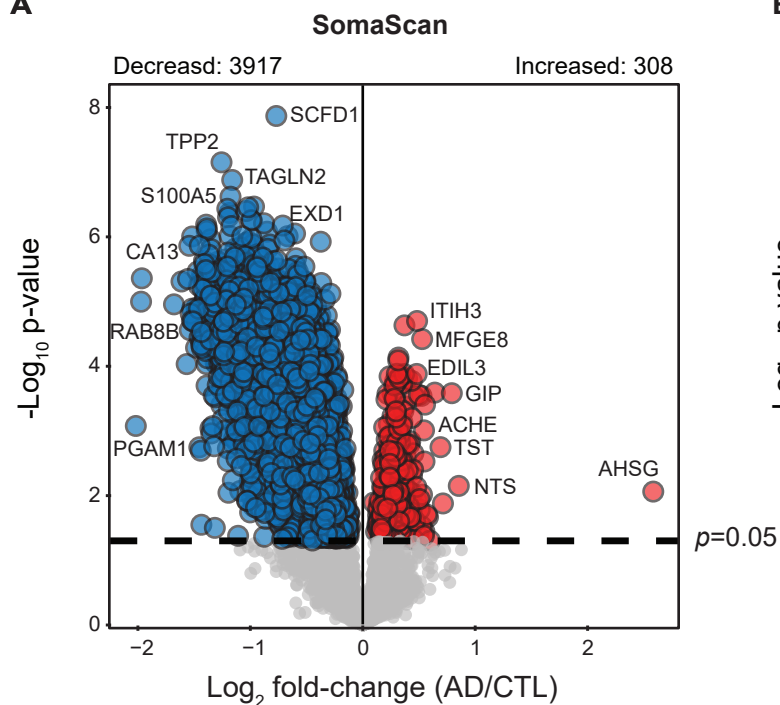**B**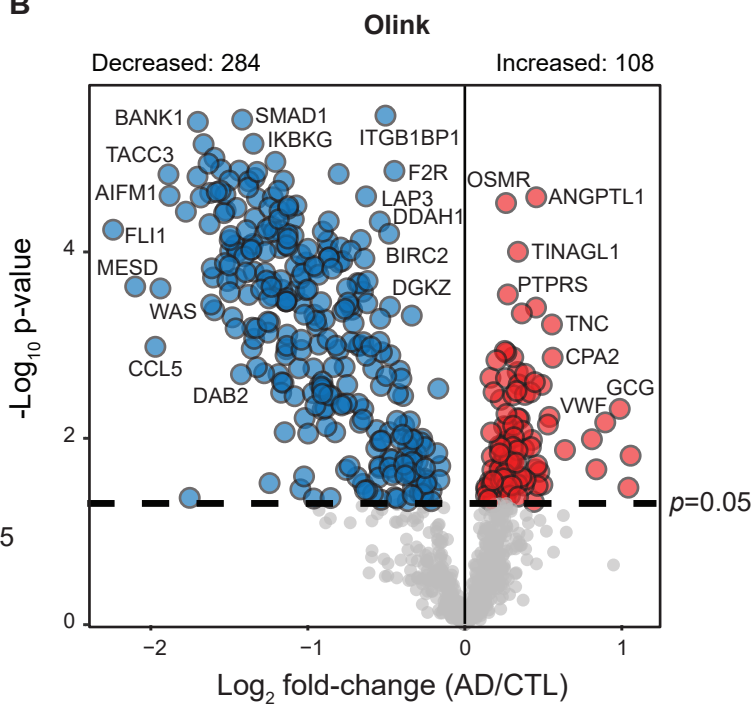**C**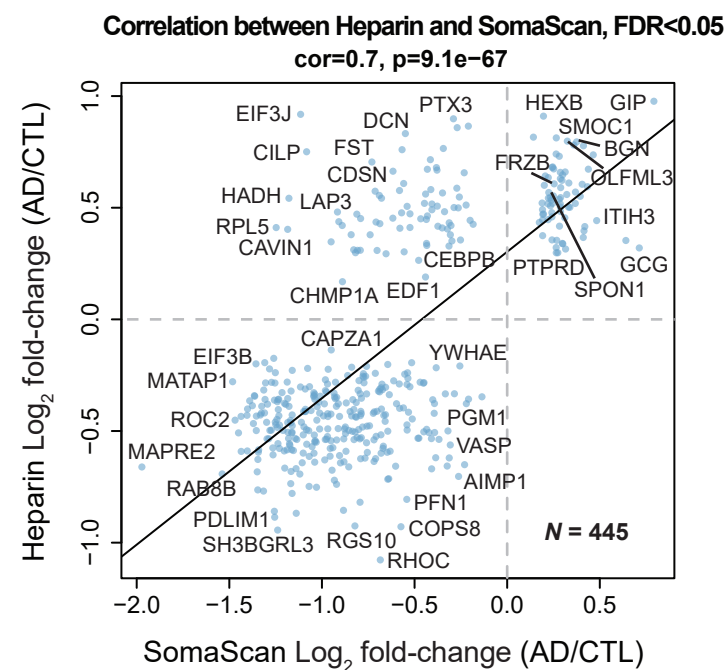**D**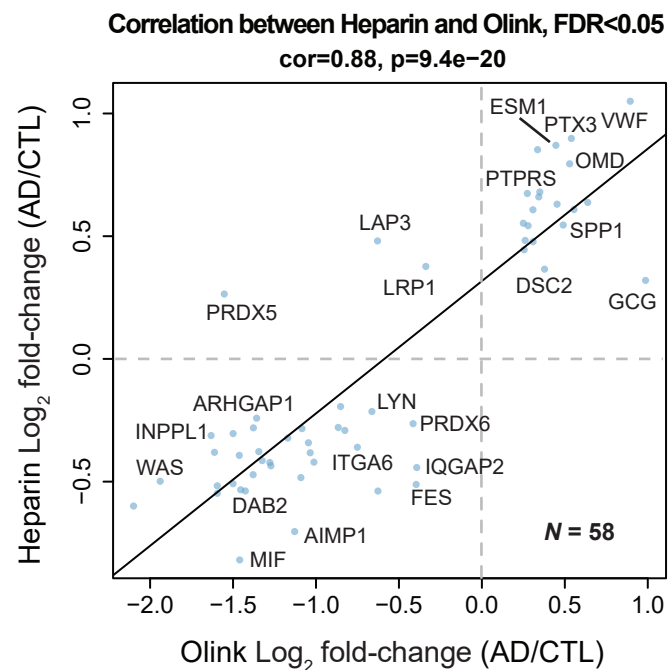

Supplement: Supplementary file 4 — Additional file 4: Supplemental Figure 4. Differential protein abundance of SomaScan and Olink and their correlations with Heparin-MS.A-B) The volcano plot illustrates the differential abundance of proteins measured by SomaScan (N = 7,284) and Olink (N = 979) between the control and AD groups. The x-axis represents the log2 fold-change (AD vs CTL), while the y-axis represents the Student’s t-statistic (-log10 p-value) calculated for all proteins in each pairwise comparison. Proteins significantly increased in AD are highlighted in red (p < 0.05), whereas those significantly decreased in AD are depicted in blue. Grey dots represent proteins with insignificant changes. For SomaScan, 308 proteins are increased in AD while 3,917 are decreased. For Olink, 108 proteins are increased in AD while 284 are decreased. Protein levels in AD are generally lower than in controls in SomaScan, resulting in a lower log2 fold-change (AD vs CTL) compared to the Heparin-MS data. C-D) Pearson correlation between log2 fold-change (AD vs CTL) of significant common gene products (FDR < 0.05) measured by the Heparin-MS and SomaScan (N = 445 gene products, cor = 0.7, p = 9.1e-67), as well as the Heparin-MS and Olink (N = 58 gene products, cor = 0.88, p= 9.4e-20). The significance of the Pearson correlation was determined by Student’s t-test. CTL, control; cor, Pearson correlation coefficient. [file 13024_2024_757_MOESM4_ESM.pdf]

**A**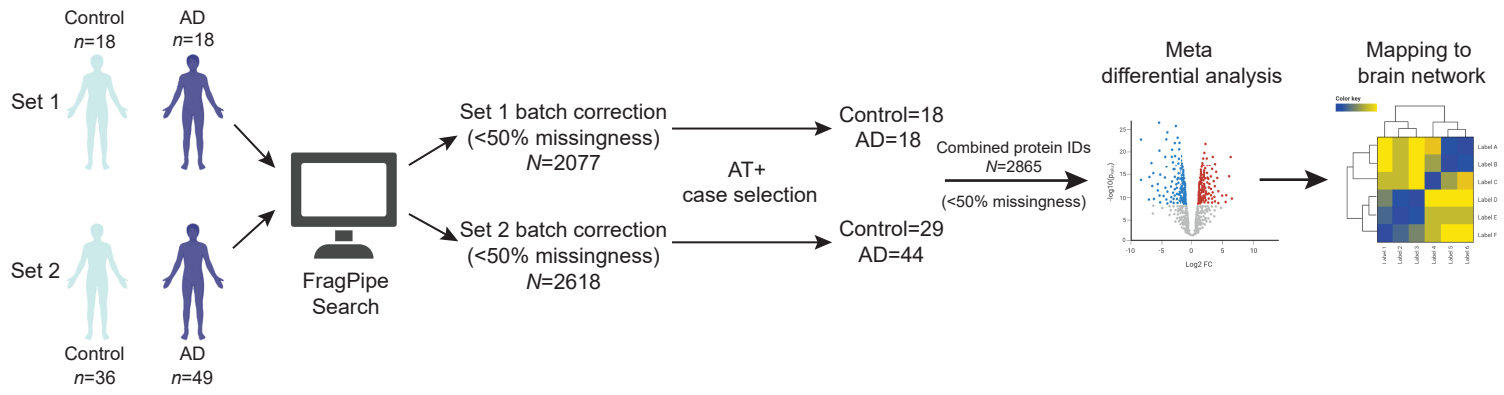**B**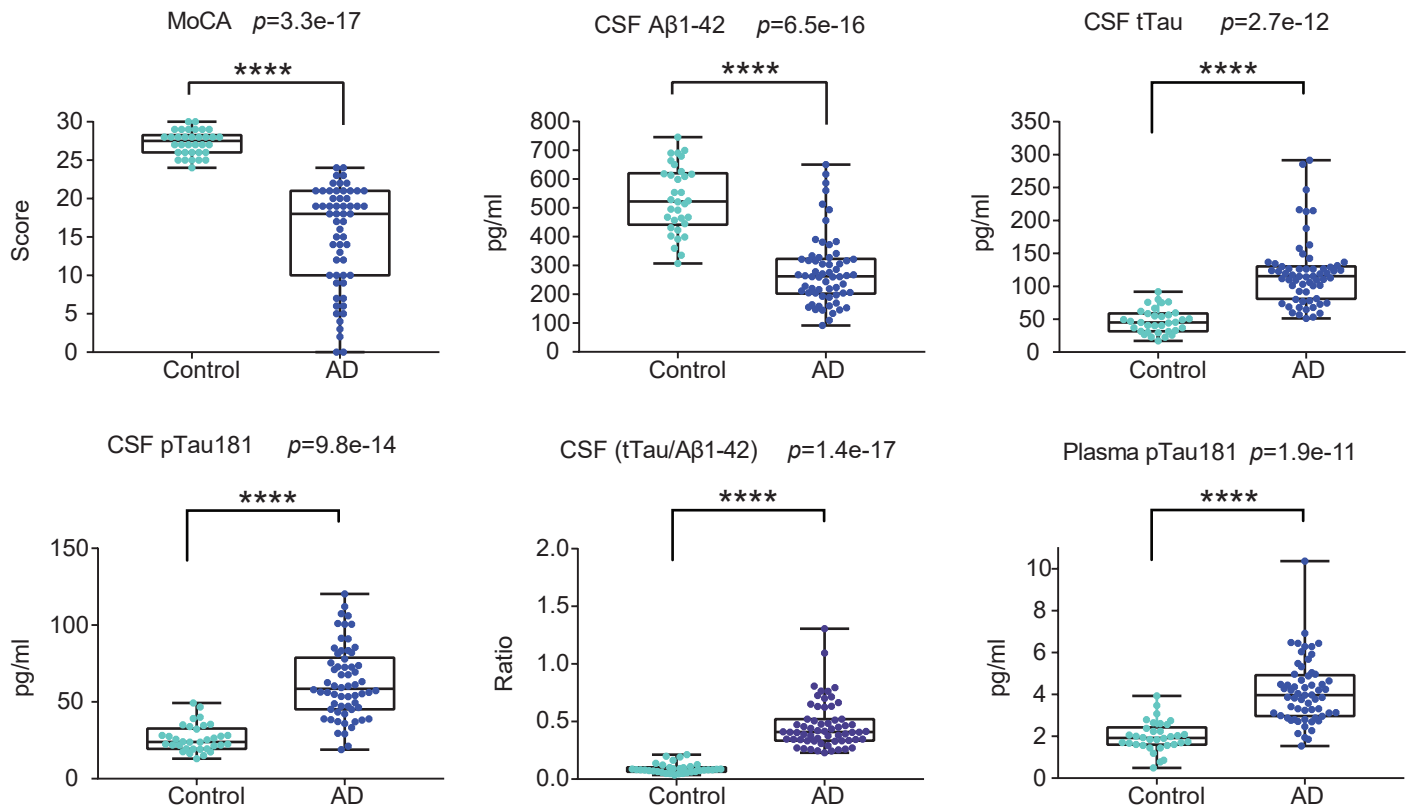

Supplement: Supplementary file 5 — Additional file 5: Supplemental Figure 5. Workflow of the data analysis pipeline across two Hp-enriched plasma datasets. A) Set 1 and Set 2 were jointly analyzed using FP, followed by independent batch-specific variance correction procedures, which included TAMPOR and batch-regression. Subsequently, 12 cases from Set 2 that did not meet the AT+ threshold criteria were excluded (see methods). A total of 109 samples and 2865 total proteins were selected for further analysis, with 13 overlapping control samples between the two datasets. B) Measurements of various AD-related traits, including cognition (MoCA score), CSF Aβ1-42, CSF tTau, CSF pTau181, CSF ratio of tTau/Aβ1-42, and plasma pTau181, were shown for the selected unique cases (n = 96). Significance levels determined by Student's t-test are denoted by overlain asterisks;*p < 0.05, **p < 0.01, ***p < 0.001, ****p< 0.0001. [file 13024_2024_757_MOESM5_ESM.pdf]

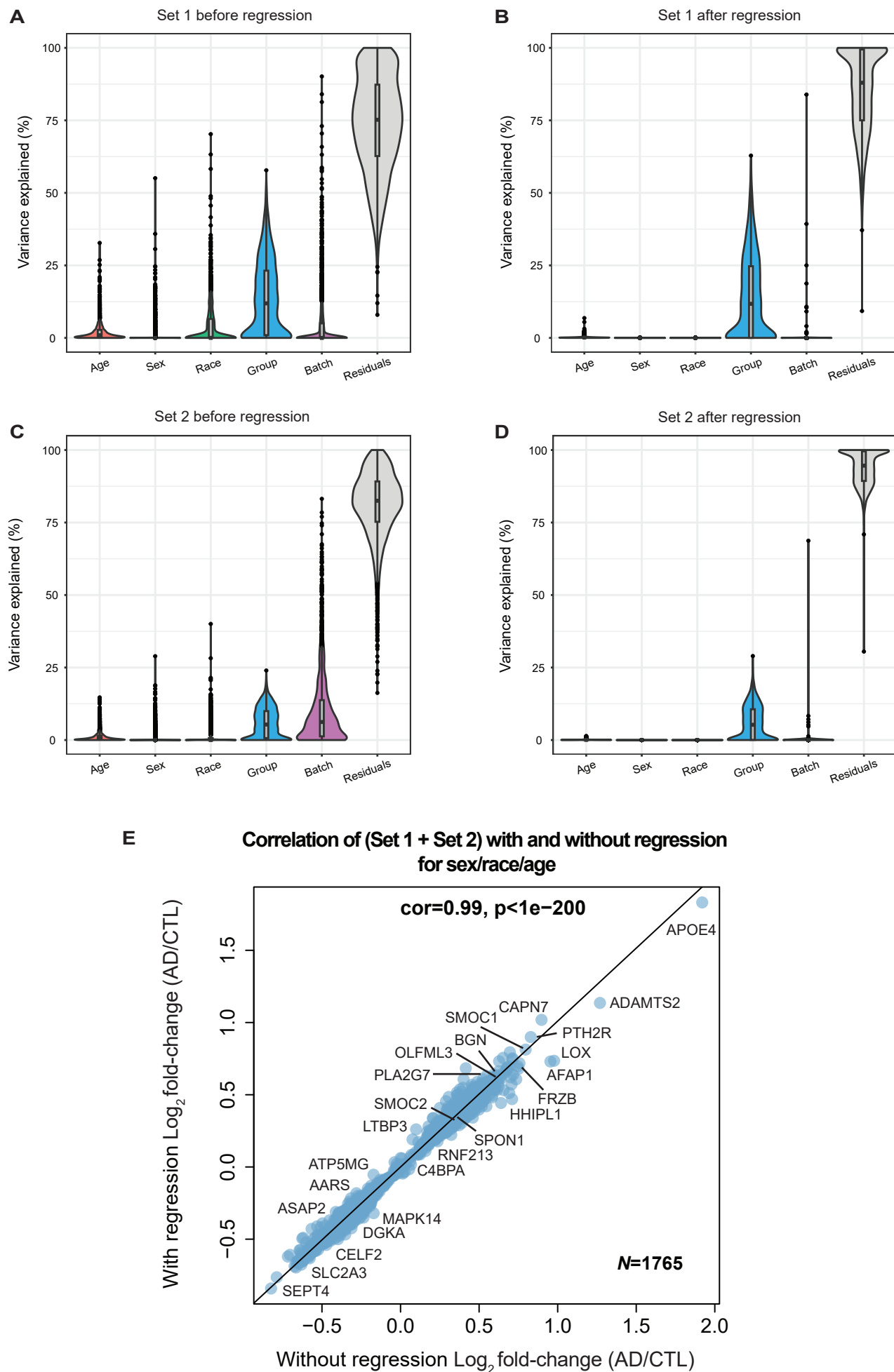

Supplement: Supplementary file 6 — Additional file 6: Supplemental Figure 6. Comparison of Set 1 and Set 2 with or without regression for age, sex, and race. A-D) Variance partition analysis is visualized by violin plots, using experimental factors to evaluate the percentage of explained variance in samples. The y-axis represents the percentage of explained variance, while the x-axis shows factors contributing to variance, including age, sex, race, group, batch, and residuals. A) Set 1 post-TAMPOR and before regression. B) Set 1 after regression. C) Set 2 post-TAMPOR and before regression. D) Set 2 after regression. Notably, variance due to age, sex, race, and batch was significantly reduced after correction, underscoring the efficacy of the correction procedure in removing trait-related variability from the proteomic data. E) A scatter plot illustrates the correlation between log2 fold-changes for AD vs CTL (cor = 0.99, p < 1e-200) on significant proteins (N = 1765, meta p < 0.05) in the meta-analysis (Set 1 + Set 2), with only batch regression or after regression for age, sex, race, and batch. CTL, control; cor, Pearson correlation coefficient. [file 13024_2024_757_MOESM6_ESM.pdf]

Supplemental Figure 8

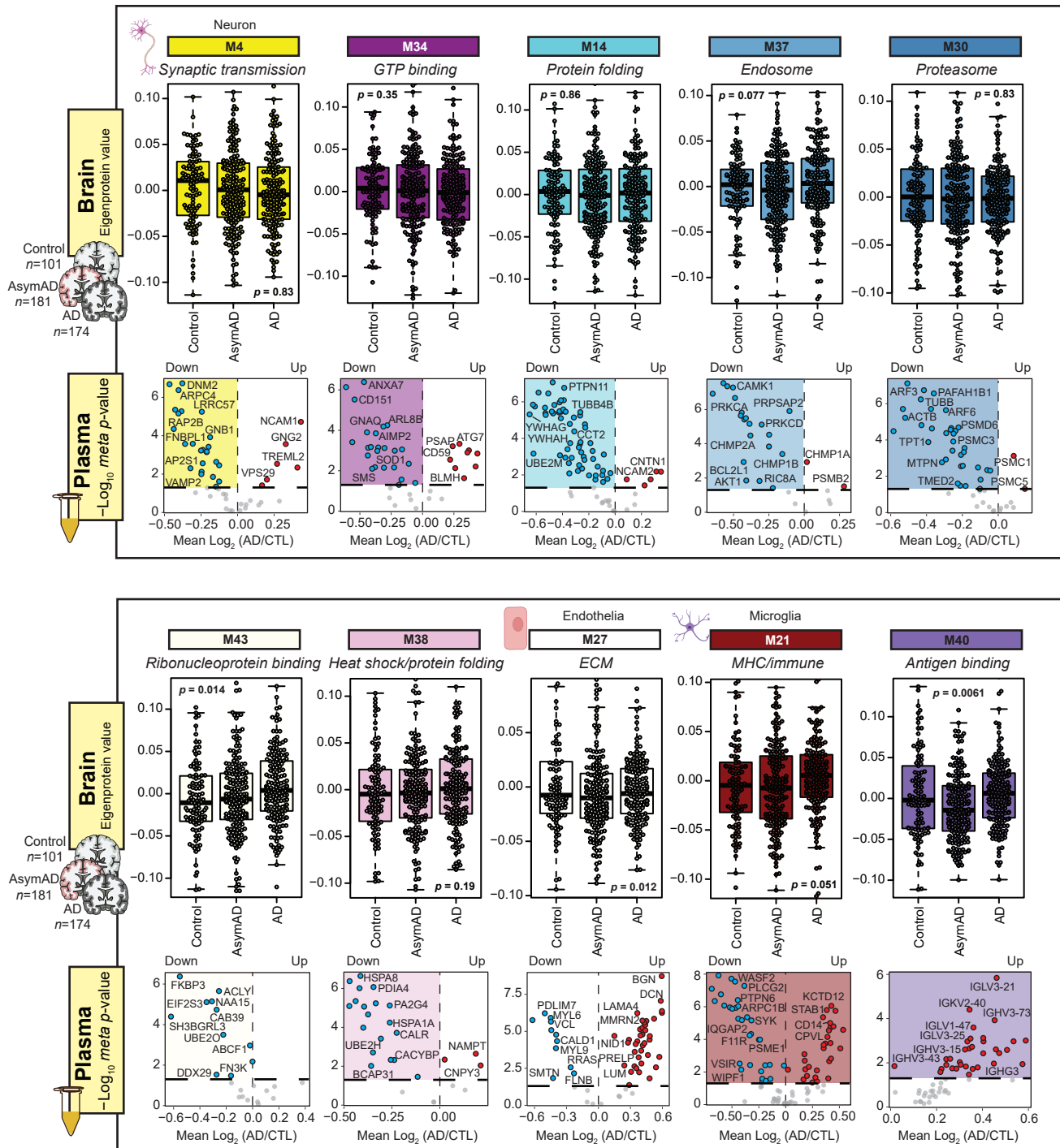

Supplement: Supplementary file 8 — Additional file 8: Supplemental Figure 8. Overlap between additional human brain network modules and differentially abundant Hp-enriched plasma proteins in AD. Protein expression trends are examined for the 10 modules that exhibit significant overlap with differentially abundant Hp-enriched plasma proteome but demonstrate moderate to low correlation with AD clinicopathological traits in the brain. Brain module abundance is quantified by eigenprotein values derived from the consensus brain dataset (11) (control = 101, AsymAD = 181, AD = 174), while volcano plots illustrate the differential abundance (log2 AD vs CTL) of module proteins overlapped with the Hp-enriched plasma proteome. The statistical significance of changes in module eigenprotein abundance across the three groups in the consensus brain cohort was assessed using ANOVA with Tukey post-hoc correction. Modules with p < 0.05 were considered significant. Among these modules, M4 ‘Synaptic transmission’, M34 ‘GTP binding’, M14 ‘Protein folding’, M37 ‘Endosome’, M30‘Proteasome’, M43 ‘Ribonucleoprotein binding’ and M38 ‘Heat shock/protein folding’ consist of proteins with decreased abundance in AD plasma, while M40‘Antigen binding’ exclusively contains increased plasma proteins in AD. M27‘ECM’ and M21 ‘MHC/immune’ exhibit a balanced representation of both increased and decreased plasma proteins in AD. CTL, control; AsymAD, asymptomatic AD. [file 13024_2024_757_MOESM8_ESM.pdf]
