# Supplementary material for: Heparin-enriched plasma proteome is significantly altered in Alzheimer’s disease
Source: Mol Neurodegener. 2024 Oct 8;19:67. doi: 10.1186/s13024-024-00757-1 (PMC11460197; doi:10.1186/s13024-024-00757-1)
Supplement: Supplementary file 7 — Additional file 7: Supplemental Figure 7. Comparing TMT-MS proteomic measurements of human brain generated by FragPipe (FP) and Proteome Discoverer (PD). A) 456 raw files collected from the ROSMAP and Banner cohorts as previously described (11) underwent a database search using FP (see methods), resulting in the identification of 8956 UniprotID-identified proteins, each with measurements available in 50% or more across 456 individual cases (control = 101, AsymAD = 181, AD = 174). These proteins were subsequently assigned to one of the 44 pre-existing consensus network modules (11) by re-calculating the kME (bicor correlation to module eigenprotein) for each protein and assigning it to the module that exhibited the highest correlation (52). B) The number and overlap of unique gene products identified in the FP and the PD outputs, with 7476 overlapping between the two datasets. FP provided an additional 1428 unique gene products compared to PD, resulting in an 18% increase in proteome coverage. C) Scatter plots illustrate the correlation between log2 fold-change for AD vs CTL (left, cor= 0.9, p < 1e-200) and AsymAD vs CTL (right, cor = 0.84, p < 1e-200), using common gene products (N = 7467) found in both FP and PD search results. CTL, control; cor, Pearson correlation of coefficient. [file 13024_2024_757_MOESM7_ESM.pdf]

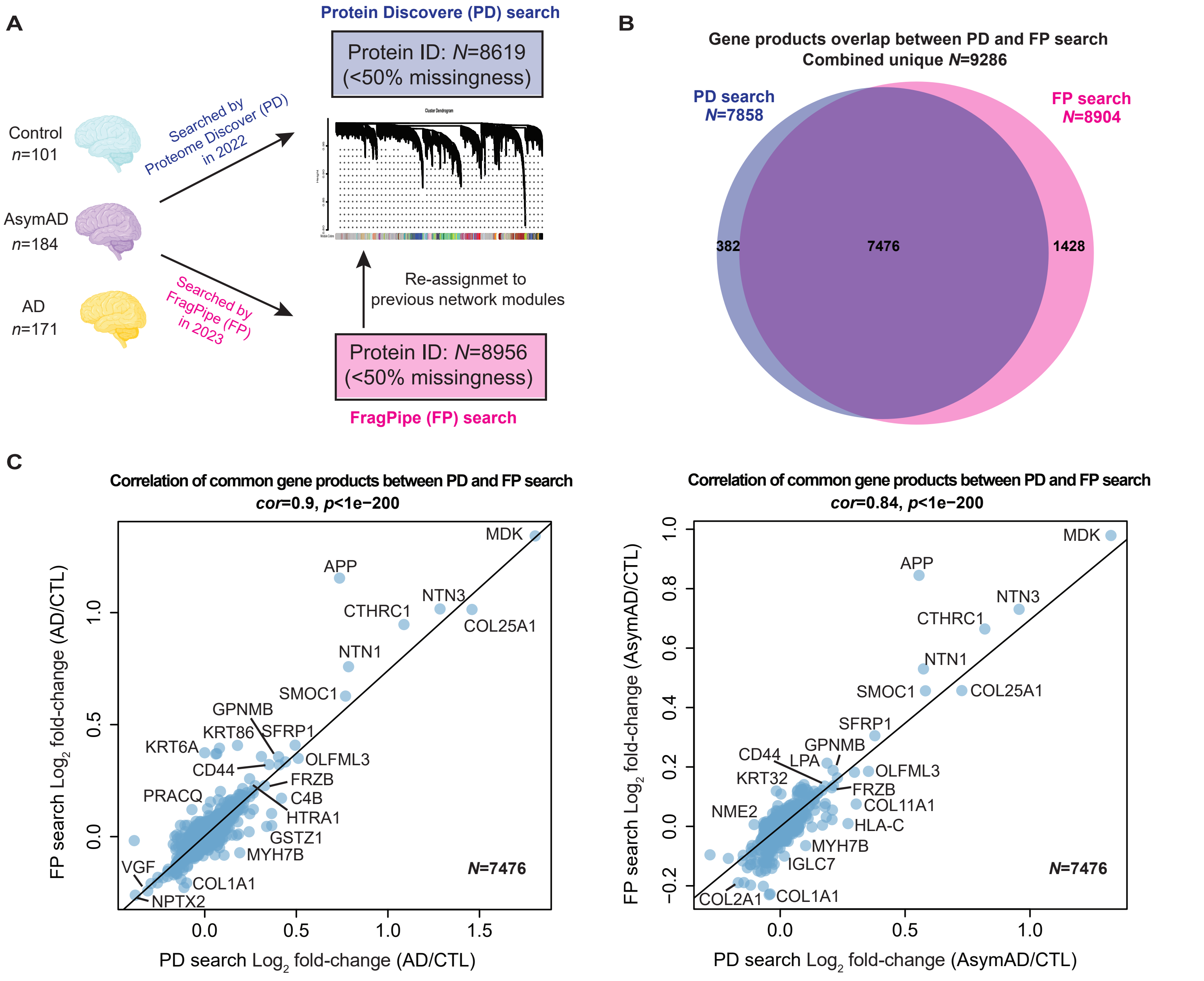
A
Protein Discovere (PD) search
Protein ID: N=8619 (<50% missingness)
Control n=101
AsymAD n=184
AD n=171
Searched by Proteome Discover (PD) in 2022
Searched by FragPipe (FP) in 2023
Cluster Dendrogram
Re-assignmet to previous network modules
Protein ID: N=8956 (<50% missingness)
FragPipe (FP) search
B
Gene products overlap between PD and FP search
Combined unique N=9286
PD search N=7858
FP search N=8904
382
7476
1428
C
Correlation of common gene products between PD and FP search
cor=0.9, p<1e−200
FP search Log2 fold-change (AD/CTL)
PD search Log2 fold-change (AD/CTL)
N=7476
MDK
APP
NTN3
CTHRC1
COL25A1
NTN1
SMOC1
GPNMB
KRT86
SFRP1
KRT6A
OLFML3
CD44
FRZB
PRACQ
C4B
HTRA1
GSTZ1
MYH7B
VGF
COL1A1
NPTX2
Correlation of common gene products between PD and FP search
cor=0.84, p<1e−200
FP search Log2 fold-change (AsymAD/CTL)
PD search Log2 fold-change (AsymAD/CTL)
N=7476
MDK
APP
NTN3
CTHRC1
NTN1
SMOC1
COL25A1
SFRP1
GPNMB
CD44
LPA
OLFML3
KRT32
FRZB
NME2
COL11A1
HLA-C
MYH7B
IGLC7
COL2A1
COL1A1
